# Delgocitinib 2% cream: a promising FDA-approved therapy for moderate-to-severe chronic hand eczema

**DOI:** 10.1097/MS9.0000000000003771

**Published:** 2025-08-27

**Authors:** Zara Jawaid, Maliha Khalid, Aminath Waafira

**Affiliations:** a Bahria University Medical and Dental College, Karachi, Pakistan; b Jinnah Sindh Medical University, Karachi, Pakistan; c School of Medicine, The Maldives National University, Malé, Maldives

**Keywords:** chronic hand eczema, delgocitinib, JAK inhibitor, topical therapy

## Abstract

Delgocitinib 2% cream (Anzupgo®) is the first U.S. FDA-approved topical Janus kinase (JAK) inhibitor for moderate-to-severe chronic hand eczema (CHE) in adults unresponsive to or unsuitable for topical corticosteroids. CHE is a multifactorial condition with significant quality-of-life impact and limited effective treatment options. Delgocitinib non-selectively inhibits JAK1, JAK2, JAK3, and TYK2, suppressing inflammatory cytokines including IFN-γ, IL-4, IL-13, IL-17A, and IL-22. Phase III randomized, double-blind, vehicle-controlled trials demonstrated a treatment success rate of up to 37.7% and a ≥4-point reduction in Hand Eczema Symptom Diary (HESD) scores after twice-daily application for 16 weeks. The cream formulation minimizes systemic risks associated with oral JAK inhibitors while providing targeted, topical relief. Adverse events are generally mild, including application site reactions, nasopharyngitis, and headache, with caution advised in pregnancy, lactation, chronic infection, and immunosuppression. Delgocitinib offers a novel, effective, and safer alternative in CHE management, warranting further research on pediatric use, long-term outcomes, and cost-effectiveness.

*To the Editor,*

This letter highlights the indications, future treatment, and outlook of Anzupgo® (delgocitinib) 2% cream, used for moderate-to-severe chronic hand eczema unresponsive to topical steroids or when steroids are not clinically indicated. Anzupgo (delgocitinib), a JAK–STAT pathway inhibitor, is the first FDA-approved therapy in the U.S. for chronic hand eczema in adults ^[1]^.

Chronic eczema (atopic dermatitis) is a non-communicable condition lasting over three months continuously or recurring at least twice annually. It is multi-etiological driven by Type I hypersensitivity (epidermal barrier dysfunction from filaggrin gene loss-of-function mutation) and Type IV hypersensitivity (T-cell-mediated elevated IgE release)^[2]^. Additional risk factors include a family history of eczema, asthma, allergic rhinitis, and increased exposure to allergens (e.g., in soaps, bleaches, dyes) or occupational wet-work. It manifests as itchy, dry, inflamed skin, particularly on flexor surfaces, inner elbows, hands, behind knees, wrists, and ankles, and affects approximately 206 million people worldwide, especially females in high-income countries^[3]^. Standard treatment strategies include allergen avoidance, antihistamines, topical corticosteroids, topical calcineurin inhibitors, and advanced biologics like dupilumab^[4]^. For severe, refractory cases, extracorporeal photopheresis (ECP) serves as an adjunct in severe cases^[5]^. However, these options may have side effects, limited efficacy in severe forms, and often require close monitoring.

Delgocitinib, is the first of its kind JAK-inhibitor which non-selectively inhibits all JAK enzymes (JAK1, -JAK2, JAK3, and TYK2) which ultimately inhibits production of inflammatory cytokines and interleukins involved such as IFN-γ, IL-4, IL-13, IL-17A, and IL-22. It provides a much significant relief from topical steroid dependency while being simultaneously targeted therapy providing symptomatic relief with safer pharmacological profile. Since it is a cream, not only it provides topical relief but also surpasses the side effects associated with oral JAK-inhibitors. This drug underwent two significant randomized, double-blind, multicenter, vehicle controlled clinical trials^[6,7]^. A treatment success rate of almost 37.7% was seen after patients were given variable doses of the drug ranging doses of 1, 3, 8, or 20 mg/g or vehicle cream, applied twice daily for 16 weeks, all with promising results. A remarkable decrease of 4 points in HESD (Hand Eczema Symptom Diary) was also seen, indicating promising improvement in quality of life, classified as the “super-responder group.” Although rendered safe, mild side effects can include application site reactions (pain, tingling, numbness), nasopharyngitis (common cold symptoms), headache and a possible leukopenia. Caution must be taken in pregnant/lactating women as safety data is less and drug must also be avoided in individuals suffering from chronic infections and immunosuppression. The FDA’s approval fills the dire need in the pharmacological landscape for chronic hand eczema (CHE). Further studies should focus on pediatric population, long term efficacy and cost-effectiveness. Our work is in line with the TITAN Guidelines on the need for transparency in AI use in healthcare^[8]^.

## Data Availability

No data were generated for this manuscript.

## Peer and provenance statement

This article was not commissioned and was externally peer-reviewed.
